# Relationship between retinopathy and renal abnormalities in black hypertensive patients

**DOI:** 10.1186/s40885-016-0053-x

**Published:** 2016-10-21

**Authors:** A. B. Omotoso, P. M. Kolo, T. O. Olanrewaju, J. F. Owoeye, S. A. Biliaminu, V. A. Olatunji

**Affiliations:** 1 Department of Medicine, University of Ilorin, P.M.B. 1515, Ilorin, Nigeria; 2 Department of Ophthalmology, University of Ilorin, P.M.B. 1515, Ilorin, Nigeria; 3 Department of Chemical Pathology, University of Ilorin, P.M.B. 1515, Ilorin, Nigeria; 4 Department of Ophthalmology, University of Ilorin Teaching Hospital, P.M.B. 1459, Ilorin, Nigeria

**Keywords:** Hypertension, Complications, Retinopathy, Kidney disease, Black Africans

## Abstract

**Background:**

Complications of hypertension are particularly common in people of African descent but previous reports had suggested rarity of hypertensive retinopathy in black Africans. We evaluated retinal changes among Nigerian hypertensive patients and determined their relationship with renal function.

**Methods:**

Consecutive Hypertensive patients who were ≥18 years were selected for the study. Socio-demographic characteristics, anthropometric parameters and blood pressure were measured. All patients had ophthalmoscopic examination and retinal changes were graded according to Keith-Wegener’s classification. Blood samples were taken for blood urea nitrogen, lipids and C-reactive protein; and urine was collected for creatinine and microalbuminuria. Serum creatinine was determined using modified Jaffe method and estimated glomerular filtration (eGFR) was calculated using MDRD equation: 186 × (Creatinine/88.4)^-1.154^ × (Age)^-0.203^ × (0.742 if female) × (1.210 if black).

**Results:**

Of the 240 patients studied, 187 (78 %) had one form of retinopathy or the other. While 85 (35.4 %) patients had grade 1 retinopathy, 87 (36.3 %) had grade 2, 13 (5.4 %) had grade 3 and 2 (0.83 %) had grade 4 respectively. Comparison of patients with and without retinopathy showed that, the former were older (*p* = 0.001) and had longer duration of hypertension (*p* = 0.001). Similarly, hypertensive patients with retinopathy had higher total cholesterol and low density lipoprotein cholesterol (*p* = 0.017, *p* = 0.041 respectively). However, eGFR was lower in individuals with retinopathy (46.2 ml/min/1.73) than those with normal retinal (55.9 ml/min/1.73) findings, *p* = 0.019. Multi-variable adjusted odds ratios showed increased probability of retinopathy with age (odds ratio-1.08, *p* = 0.001) and body mass index (odds ratio-1.20, *p* = 0.013).

**Conclusions:**

Hypertensive retinopathy is a common clinical finding among hypertensive Nigerians and may occur *pari passu* with renal damage as consequences of long standing hypertension.

## Background

Systemic hypertension is a major public health concern that affects more than one billion people world-wide [1]. The prevalence of hypertension is known to be high in black Africans in whom the disease presents early and runs a rapid course [2]. Retinal micro-vascular abnormalities such as arterial narrowing, arteriovenous nicking and retinopathy reflect the vascular damage from hypertension [3]. These retinal changes appear to be irreversible long term markers of hypertension and may relate to current and past blood pressures. The presence of retinopathy indicates that a person’s hypertension has progressed to a severe stage with associated target organ damage [4]. Blood vessel damage in the eye as a result of hypertension has been tracked with similar changes in the brain and has been found to be a potential risk of stroke and death, independent of known risk factors [5]. The presence of retinopathy may be an indication for initiating antihypertensive treatment, even in people with stage 1 hypertension (blood pressure, 140 to 159/90 to 99 mm Hg) who have no other evidence of target-organ damage [6]. Although, an earlier study among continental Africans [7] showed hypertensive retinopathy to be rare, evidence is accumulating to suggest that the incidence of hypertensive retinopathy is higher among Africans than Caucasians in United States of America [8, 9]. Blacks are also more likely to develop hypertension-related complications such as heart failure, stroke and kidney diseases than Whites. Indeed, the combination of target organ damage (left ventricular hypertrophy (LVH) and chronic kidney disease) in patients with systemic hypertension has been found to exaggerate risk of cardiovascular events [10]. The involvement of retina in hypertension is similar to the blood vessel damage in the kidneys (arteriosclerosis) but whether this occur simultaneously in the course of the disease is not very clear. We therefore evaluated retinal and renal profiles in hypertensive patients in our hospital.

## Methods

The study was a descriptive cross sectional evaluation of retinal morphology and renal function among hypertensive patients that were managed at the Medical Out-Patient Department (MOPD) of our hospital. Consecutive hypertensive patients seen at the MOPD were recruited if they were ≥18 years and gave informed consent. Hypertensive patients, who were diabetic, had sickle cell anaemia, had history of glaucoma, pregnant and those with secondary hypertension were excluded from the study. Ethical approval was obtained from the Ethics and Research Committee of the University of Ilorin Teaching hospital, Nigeria. The socio-demographic characteristics of the participants were obtained and a thorough history including the duration of hypertension, drug therapy and compliance was taken. The anthropometric parameters, blood pressure and other relevant clinical examination were performed on the patients. Blood pressure was measured at the left arm in a comfortable sitting position after about 10 min rest using standard mercury sphygmomanometer (Accoson) with an appropriate cuff. However, if the blood pressure was equal or greater than 10 mmHg at the right arm, the latter blood pressure was used as the blood pressure of the subjects. An average of three measurements was taken as the patient’s blood pressure. Hypertension was defined according to the JNC VII (BP ≥ 140/90 mmHg or anti-hypertensive use) [11]. The intra-ocular pressure was measured using Perkins applanation tonometer. All patients had ophthalmoscopic examination done by one of the ophthalmologists (OVA) on the team after pupillary dilatation with 0.5 % cyclopentolate eye drops or 1 % tropicamide and retinal changes compatible with hypertensive changes were assessed. The severity of patient’s retinal changes was determined and graded according to Keith-Wegener’s classification. When in doubt the opinion of the second ophthalmologist (JFO, more experienced) was sort who also intermittent examine sample of patients for concordance. Blood samples were taken from each patient for blood urea nitrogen, lipids and C-reactive protein; and urine was collected for creatinine and microalbuminuria. Serum Blood urea nitrogen was determined using Modified Berthelot’s method. Serum C-reactive protein was determined using Turbilatex method with high sensitivity and specificity. Serum estimation of high density lipoprotein cholesterol (HDL-c) and low density lipoprotein cholesterol (LDL-c) were done using Direct Immunoinhibition method. Serum Total Cholesterol and Triglycerides were determined using colorimetric enzymatic method. Urine Microalbumin was determined using quantitative Turbidimetric Immunoassay method. Albumin creatinine ratio (ACR) was calculated using the formula: Urine Albumin (mg/dL)/Urine Creatinine (g/dL) = ACR in mg/g ≈ Albumin excretion in mg/day. Serum Creatinine was determined using modified Jaffe method and eGFR was calculated using MDRD equation: 186 × (Creat/88.4)^-1.154^ × (Age)^-0.203^ × (0.742 if female) × (1.210 if black). Where unit of eGFR = mL/min/1.73 m^2^, serum creatinine = μmol/L and age = years. Serum Uric Acid was determined using Uricase-PAP method. All the kits used were manufactured by Agappe Diagnostics International and supplied by NUMS Diagnostics Nigeria Ltd. Suleja.

### Data analysis

Statistical analysis was performed using the SPSS Version 15 (SPSS Inc, Chicago Illinois, USA 2006 edition) and the numerical values were presented as mean ± SD. Student t-test was used to compare means of continuous variables while chi-square test was used to compare means of proportions. Means of variables of interest in patients with and without retinopathy; and those with ACR ≤300 mg/g and >300 mg/g were compared using independent t-test. Relationship between retinopathy and other variables of interest were determined by Spearman’s Rank correlation method. Those variables that have no significant correlations with retinopathy were excluded from analysis and the strength of association of each of the remaining variables was assessed using partial correlation statistics. Binary logistic regression was used to determine predictors of retinopathy and the products were expressed as odd ratio with 95 % confident interval. A statistically significant association was set at *P* < 0.05.

## Results

Two hundred and forty hypertensive patients comprising of 160 (66.7 %) women and 80 (33.3 %) men were studied with mean age of 58.9 ± 12.1 years. Baseline clinical and biochemical characteristics of the patients are shown in Table 1. The mean BMI in the study participants was 26.6 ± 5.8 kg/m^2^ which is in the over-weight category. The mean total cholesterol was 5.9 ± 1.5 mmol/l) while the mean of HDL-c was 0.9 ± 0.2 mmol/l with coronary artery disease risk ratio of 6.6. Median of the eGFR was 49.3 (range 16.5–167.2) mL/min/1.73 m^2^ which suggests that significant number of the patients had reduced kidney function. Similarly, the median of ACR was 93.9 (3.3–1931.3 mg/g) which also suggests that a good number of subjects had significant albuminuria.

**Table 1:** ​Baseline characteristics of the patients (*n* =240). Characteristics are presented as mean ± SD, median (range)

Gender (Male/Female), n(%)	80/160 (33.3/66.7)
Age (years)	58.9 ± 12.1
Duration of hypertension (years)	6 (1–37)
Body Mass Index (Kg/m^2^)	26.6 (5.8)
Systolic Blood pressure (mmHg)	139.86 ± 22.1
Mean Arterial Pressure (mmHg)	104.1 ± 15.0
Pulse Pressure (mmHg)	53.3 ± 16.5
Diastolic Blood Pressure (mmHg)	86.3 ± 13.6
Mean Blood Pressure(mmHg)	97.4 ± 7.9
Waist Circumference (m)	97.4 ± 14.4
Hip Circumference (m)	101.7 ± 14.1
Waist Hip Ratio	0.97 ± 0.11
Uric Acid (mmol/L)	7.5 ± 3.4
Total cholesterol (mmol/L)	5.9 ± 1.5
HDL-Cholesterol (mmol/L)	0.9 (0.2)
Triglyceride (mmol/L)	2.2(0.9)
Low Density Lipoprotein (mmol/L)	2.8 ± 1.0
C-Reactive Protein (g/L)	7.8 ± 2.8
Albumin-Creatinine Ratio (mg/g)	93.9 (3.3–1931.3)
eGFR by MDRD (ml/min/1.73)	49.3 (16.5–167.2)

**Fig. 1:**
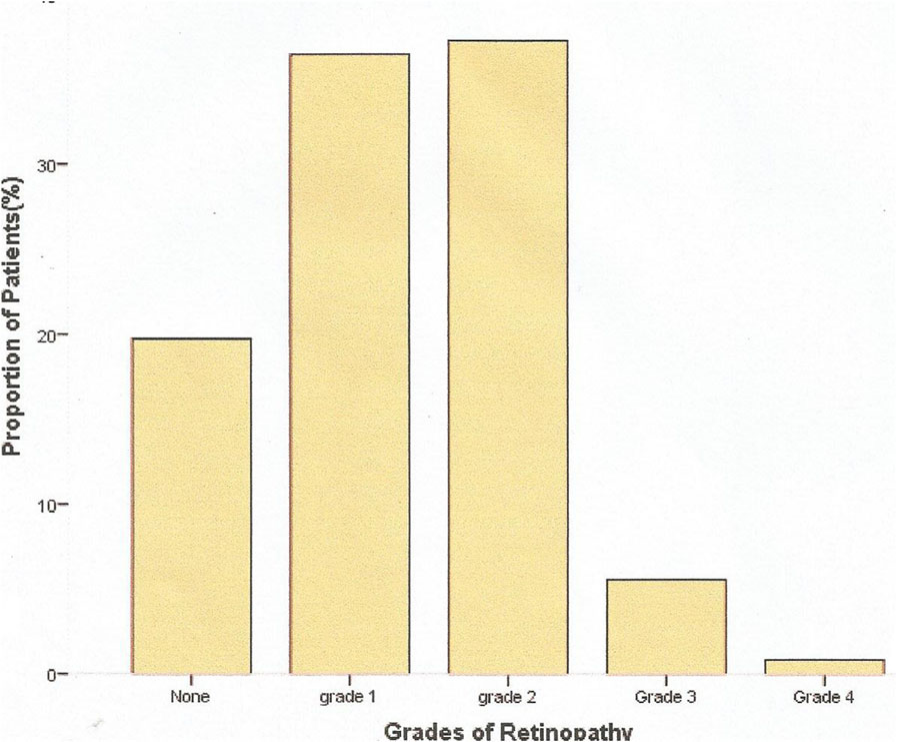
shows frequency of hypertensive retinopathies among the participants

Figure 1 shows frequency of hypertensive retinopathies among the participants. One hundred and eighty seven patients (78 %) had one form of retinopathy or the other. While 85 (35.4 %) patients had grade 1 retinopathy, 87 (36.3 %) had grade 2, 13 (5.4 %) had grade 3 and 2 (0.83 %) had grade 4 respectively.

When patients with hypertensive retinopathy were compared with those without retinopathy, the former were older (*p* = 0.001) and had longer duration of systemic hypertension (*p* = 0.001) as shown in Table 2. Similarly, hypertensive patients with retinopathy have higher total cholesterol and low density lipoprotein cholesterol (*p* = 0.017, *p* = 0.041 respectively). On the other hand, eGFR was lower in individuals with hypertensive retinopathy (46.2 ml/min/1.73) compared with those with normal retinal (55.9 ml/min/1.73) findings, *p* = 0.019. However, body mass index, blood pressure indices, C-reactive protein and ACR were similar between patients with and without retinopathy. Similarly, patients on drug combinations that included angiotensin converting enzyme inhibitors (ACEI) were compared with those on combinations without ACEI. All parameters compared were similar except ACR which was lower in those on ACEI than those not on ACEI. The median of ACR was 77.3 (5.78–1931.3 mg/g) in the former and 104.6 (3.34–1706.3 mg/g) in the latter. In addition, fewer patients on ACEI (40.4 %) have grades II-IV retinopathy compared with those not on ACEI (46.4 %), *p* = 0.346.

**Table 2:** Characteristics of patients with and without retinopathy. Characteristics are presented as mean ± SD, median (range)

	Patients with retinopathy (*n* = 187)	Patients without retinopathy (*n* = 46)	*P* value
Gender (Male/Female), n(%)	80/160 (33.3/66.7)		
Age (years)	61.7 ± 10.5	47.2 ± 12.0	0.001
Duration of hypertension (years)	7 (1–37)	4.5 (1–15)	0.001
Body Mass Index (Kg/m^2^)	26.9 ± 6.0	26.1 ± 4.9	0.422
Systolic Blood pressure (mmHg)	139.5 ± 22.6	141.2 ± 20.8	0.657
Diastolic Blood Pressure (mmHg)	85.8 ± 14.0	88.8 ± 11.8	0.183
Mean Blood Pressure(mmHg)	103.7 ± 15.4	106.2 ± 13.9	0.307
Pulse Pressure (mmHg)	53.8 ± 17.2	52.4 ± 14.2	0.307
Waist Circumference (m)	98.0 ± 14.7	95.6 ± 13.8	0.321
Hip Circumference (m)	102.3 ± 13.9	99.9 ± 14.8	0.308
Waist Hip Ratio	0.97 ± 0.11	0.96 ± 0.13	0.751
Uric Acid (mmol/L)	7.5 ± 3.4	7.5 ± 3.4	0.966
Total cholesterol (mmol/L)	6.1 ± 1.4	5.4 ± 1.7	0.017
HDL-Cholesterol (mmol/L)	0.93 ± 0.21	0.91 ± 0.21	0.593
Triglyceride (mmol/L)	2.3 ± (1.0)	2.0 ± 0.8	0.130
Low Density Lipoprotein (mmol/L)	2.9 ± 1.0	2.5 ± 1.2	0.041
C-Reactive Protein (g/L)	7.9 ± 2.8	7.2 ± 3.1	0.116
Albumin-Creatinine Ratio (mg/g)	93.9(3.9–1931.3)	109.6(3.3–1580)	0.908
eGFR by MDRD (ml/min/1.73)	46.2(16.5–167.2)	55.9(22.5–165.4)	0.019

Comparison of patients with ACR ≤300 mg/g against those who had ACR >300 mg/g is presented in Table 3. The mean age and duration of hypertension were similar between the former and the latter. Patients with ACR > 300 mg/g have higher percentage of grade II-IV retinopathy, serum uric acid level, total cholesterol, low density lipoprotein cholesterol, triglycerides and C-reactive protein than those with ACR ≤ 300 mg/g. On the other hand, those with ACR > 300 mg/g have lower high density lipoprotein cholesterol and eGFR.

**Table 3:** Characteristics of patients with ACR equal or less than 300 mg/g and greater than 300 mg/g Characteristics are presented as mean ± SD, median (range) or number (%)

	Patients with ACR ≤300 mg/g (*n* = 167)	Patients with ACR > 300 mg/g (*n* = 62)	*P* value
Gender (Male/Female), n(%)	80/160 (33.3/66.7)		
Age (years)	58.6 ± 11.4	57.5 ± 12.8	0.548
Duration of hypertension (years)	7.8 ± 7.1	7.8 ± 6.6	0.979
Body Mass Index (Kg/m^2^)	26.2 ± 5.3	27.9 ± 6.8	0.060
Systolic Blood pressure (mmHg)	139.6 ± 20.8	137.2 ± 20.1	0.470
Diastolic Blood Pressure (mmHg)	86.1 ± 13.2	86.3 ± 12.5	0.895
Mean Blood Pressure(mmHg)	103.9 ± 14.1	103.3 ± 13.7	0.783
Pulse Pressure (mmHg)	53.6 ± 16.6	50.9 ± 15.2	0.309
Waist Circumference (m)	96.9 ± 12.0	98.1 ± 19.5	0.579
Hip Circumference (m)	101.5 ± 12.9	103.4 ± 14.4	0.380
Waist Hip Ratio	0.96 ± 0.11	0.96 ± 0.08	0.882
Uric Acid (mmol/L)	7.2 ± 3.0	8.6 ± 4.4	0.017
Total cholesterol (mmol/L)	5.7 ± 1.5	6.9 ± 1.1	0.001
HDL-Cholesterol (mmol/L)	0.95 ± 0.23	0.89 ± 0.13	0.040
Triglyceride (mmol/L)	2.1 ± (1.0)	2.7 ± 0.9	0.001
Low Density Lipoprotein (mmol/L)	2.7 ± 1.0	3.4 ± 0.8	0.001
C-Reactive Protein (g/L)	7.5 ± 2.7	9.2 ± 2.2	0.001
Retinopathy (II-IV) Frequency%	34 (58.6 %)	63 (42.3 %)	0.0316
eGFR by MDRD (ml/min/1.73)	58.8 ± 28.9	50.3 ± 19.2	0.021

Correlates of hypertensive retinopathy are shown in Table 4. Significant positive correlations were observed between age, duration of hypertension, body mass index, total cholesterol, LDL-c and C-reactive protein with retinopathy. On the other hand, significant negative correlation was observed between eGFR and retinopathy. However, when each of the parameters were assessed individually (partial correlation), the correlation of total cholesterol and C-reactive protein with retinopathy were no longer significant. Binary logistic regression to determine predictors of retinopathy is presented in Table 5. Multi-variable adjusted odds ratios showed increased probability of retinopathy with age (odds ratio-1.08, *p* = 0.001) and body mass index (odds ratio-1.20, *p* = 0.013)

**Table 4:** Association of retinopathy with clinical and laboratory characteristics

Characteristic	Correlation coefficient	*P* value
Age (years)	0.41	0.001
Duration of hypertension (years)	0.29	0.001
Body Mass Index (Kg/m^2^)	0.16	0.017
Systolic Blood pressure (mmHg)	0.07	0.295
Diastolic Blood Pressure (mmHg)	−0.05	0.946
Mean Blood Pressure(mmHg)	−0.03	0.637
Pulse Pressure (mmHg)	0.10	0.145
Waist Circumference (m)	0.09	0.200
Hip Circumference (m)	0.04	0.574
Waist Hip Ratio	0.08	0.226
Uric Acid (mmol/L)	−0.001	0.990
Total cholesterol (mmol/L)	0.22	0.002
HDL-Cholesterol (mmol/L)	−0.01	0.885
Low Density Lipoprotein (mmol/L)	0.23	0.001
Triglyceride (mmol/L)	0.11	0.111
Albumin-Creatinine Ratio (mg/g)	0.002	0.974
C-Reactive Protein (g/L)	0.17	0.016
eGFR by MDRD (ml/min/1.73)	−0.26	0.001

**Table 5:** Multi-variable adjusted odds ratio for probability of retinopathy

Variables	Odd Ratio	95 % CI	*P*-value
Age	1.08	1.044–1.127	0.001*
DOH	1. 05	0.996–1.115	0.067
BMI	1.20	1.040–1.389	0.013*
SBP	1.01	0.987–1.031	0.448
DBP	1.02	0.984–1.055	0.300
ACR	1.00	1.000–1.002	0.211
eGFR	1.00	0.969–1.005	0.146

Key: *CI* confidence interval, *DOH* duration of hypertension, *BMI* body mass index, *SBP* systolic blood pressure, *DBP* diastolic blood pressure, *ACR* albumin creatinine ratio, estimated glomerular filtration rate

*statistically significant

## Discussion

Systemic hypertension remains the most prevalent cardiovascular risk factor in indigenous African population and a major cause of cardiovascular events. This prospective cross sectional study evaluated common complications of the eye and the kidneys among hypertensive individuals. The prevalence of hypertensive retinopathy among the study participants was 78 %. This prevalence is comparable with that of Ladipo [8] who reported a prevalence of over 70 % among Nigeria hypertensive patients. Our result is also similar to prevalence of retinopathy reported among black hypertensive patients in other African countries [9, 12, 13], but higher than results among hypertensive patients in Europe [14] and blacks in United States of America [15]. On the other hand, our finding contrasts that of Akinkugbe [7] who reported that hypertensive retinopathy was a rare occurrence among African patients. Perhaps, patients’ selection and sample size may account for this difference as Akinkugbe’s study was limited by small sample size and heterogeneous nature of his sample population. Complications of hypertension such as LVH, stroke and kidney failure have been reported to be commoner among Blacks than the Whites; and retinal changes may not be an exception as shown in our study. The high prevalence of retinopathy (an indication of changes in the cerebral arteries) among study participants may also explain why there is high prevalence of stroke in our hypertensive population [16].

Patients who had retinopathy are older than those with normal retinal examination and positive correlation was observed between age and retinopathy. Age has been demonstrated as an independent predictor of retinopathy in patients with hypertension in other studies [17, 18]. Age together with other factors such as duration of hypertension, BMI, total cholesterol, LDL-c and C-reactive protein correlated with retinal changes in our patients. The clustering of cardiovascular risk factors in the patients studied could account for high prevalence of hypertensive retinopathy.

Blood pressure indices (systolic and diastolic blood pressures) have been found to be associated with risk of development of hypertensive retinal changes [19, 20]. This is because hypertensive retinopathy is a reflection of severity of blood pressure elevation and control over a period of time. However, systolic and diastolic blood pressures were similar between patients with retinopathy and those without in our study.

Analysis of renal function among the study participants showed that eGFR was lower (46.2 ml/min/1.73) in those with retinopathy than those without (55.9 ml/min/1.73), *p* = 0.019. Similarly, a significant negative correlation was observed between the presence of retinopathy and eGFR (*R* = −0.26, *P* = 0.001). These results show that a significant number of our hypertensive patients had reduction in their renal function and this may occur simultaneous with other end organ consequence of the disease including retinal changes. In addition, ACR showed that a significant number of the patients studied had albuminuria which is an indication of glomerular dysfunction [21]. When patients with ACR ≤ 300 mg/g were compared with those with ACR >300 mg/g, the latter have higher percentage of grades II-IV retinopathy, higher levels of serum uric acid, C-reactive protein and dyslipidaemia than the former. This is an indication of a more severe disease among those with ACR > 300 mg/g than those with ACR ≤300 mg/g. Elevated serum uric acid level is associated with increased risk of chronic kidney disease [22]. Long standing hypertension is associated with vascular changes (arteriosclerosis) which occur in all organs of the body including the eyes. Therefore, the presence of hypertensive retinopathy detected at fundoscopy may be an indication for a more extensive evaluation of function of other target organs in hypertension such as the kidneys [23].

Multi-variable adjusted odds ratios showed increased probability of retinopathy with age (odds ratio-1.08, *p* = 0.001) and body mass index (odds ratio-1.20, *p* = 0.013). Obesity is considered to be a major cardiovascular risk factor and microalbuminuria is an indication of renal disease. Indeed, retinopathy, LVH and microalbuminuria are considered as evidence of target organ damage in systemic hypertension. Our study further confirmed the beneficial role of ACEIs in the management of systemic hypertension [24]. It is worthy of note that patients on drug combinations including ACEIs had lower ACR compared with those not on this drug. ACEIs protect the kidneys and can prevent or delay onset of proteinuria in kidney disease.

This study was limited by over representation of women in our study participants which is a reflection of health seeking behaviours among hypertensive patients seen in our practice. Therefore, it may be difficult to generalize our result to our population. Secondly, direct ophalmoscopy was employed in the assessment of the retina in our patients. Although, this is not inferior to retinal photographs but might have provided opportunity for further reviews.

## Conclusion

Hypertensive retinopathy is a common clinical finding among hypertensive Nigerians and occurs *pari passu* with renal damage as consequences of long standing hypertension. The presence of hypertensive retinopathy may be an indication for further assessment of affected individual especially the renal function.

## Abbreviations

ACEI: Angiotensin converting enzyme inhibitor
ACR: Albumin creatinine ratio
BMI: Body mass index
eGFR: Estimated glomerular filtration rate
HDL-c: High density lipoprotein cholesterol
JNC: Joint National Committee
LDL-c: Low density lipoprotein cholesterol
LVH: Left ventricular hypertrophy
MDRD: Modification of diet in renal disease
MOPD: Medical out-patient department
